# Immunological Role and Prognostic Value of APBB1IP in Pan-Cancer Analysis

**DOI:** 10.7150/jca.50785

**Published:** 2021-01-01

**Authors:** Qianyun Ge, Ganxun Li, Jin Chen, Jia Song, Guangzhen Cai, Yi he, Xuewu Zhang, Huifang Liang, Zeyang Ding, Bixiang Zhang

**Affiliations:** 1 Hepatic Surgery Center, Tongji Hospital, Tongji Medical College, Huazhong University of Science and Technology, Wuhan, China.; 2 Hubei Key Laboratory of Hepato-Pancreato-Biliary Diseases, Tongji Hospital, Tongji Medical College, Huazhong University of Science and Technology, Wuhan, China.

**Keywords:** APBB1IP, pan-cancer analysis, immune infiltration, prognosis, tumor immune microenvironment

## Abstract

**Objective:** APBB1IP is a Rap1-binding protein that mainly acts as a regulator of leukocyte recruitment and pathogen clearance through complement-mediated phagocytosis. However, the role of *APBB1IP* in tumor immunity remains unclear. This study was carried out to evaluate the prognostic landscape of *APBB1IP* in pan-cancer analysis and investigate the relationship between *APBB1IP* expression and immune infiltration.

**Methods:** We explored the expression pattern and prognostic value of *APBB1IP* in pan-cancer analysis through Kaplan-Meier Plotter and multiple databases, including TCGA, Oncomine. We then assessed the correlation between *APBB1IP* expression and immune cell infiltration using the TIMER database. Furthermore, we identified the proteins that interact with APBB1IP and performed epigenetic and transcriptional analyses. Multivariate Cox regression analyses were applied to construct a prognostic model, which consisted of APBB1IP and its interacting proteins, based on the lung cancer cohorts from the Gene Expression Omnibus (GEO) database.

**Results:** The expression of APBB1IP was correlated with the prognosis of several types of cancer. APBB1IP upregulation was found to be associated with increased immune cell infiltration, especially for CD8^+^ T cells, natural killer (NK) cells, and immune regulators. A link was found between APBB1IP and immune-related proteins including RAP1A/B, TLN1/2 and VCL in the interaction network.

**Conclusion:** APBB1IP can serve as a prognostic biomarker in pan-cancer analysis. APBB1IP upregulation was correlated with increased immune-cell infiltration, and the expression APBB1IP in different tumors might be related to the tumor immune microenvironment.

## Introduction

The amyloid β (A4) precursor protein-binding, family B, member 1 interacting protein (*APBB1IP*) was identified as a binding partner of the amyloid β (A4) precursor protein-binding, family B, member 1 (*APBB1*) 1. Subsequently, *APBB1IP* was found to interact with the small guanosine triphosphatase (*GTPase*) *Rap1* in a yeast two-hybrid screen 2. *APBB1IP* belongs to the *MRL* (*Mig-10*/*RIAM*/*Lamellipodin*) family of adaptor proteins, which have a proline-rich region at the C terminus and a highly conserved pattern of 27 amino acids in a predicted coiled-coil region immediately N-terminal to the RA domain 2.

*APBB1IP* is an intrinsic element of the integrin activation machinery, and is required for Rap1-induced affinity changes in β1 and β2 integrins in T cells 2. Moreover, *APBB1IP* was demonstrated to also be involved in Rap1-mediated activation of αIIbβ3 integrin in platelets 3. Based on these molecular mechanisms, the *APBB1IP* mainly is thought to mainly function in the activation and modulation of innate immune responses, as a regulator of leukocyte recruitment and pathogen clearance through complement-mediated phagocytosis 4-6. However, *APBB1IP* also plays a central role in cancer cell migration and invasion, and *APBB1IP*-depleted melanoma cells displayed decreased persistent cell migration directionality, thereby reducing cancer invasion 7. However, there are no systemic studies on the role of *APBB1IP* in different human cancers, and it remains unclear whether the effects of *APBB1IP* might be context-dependent, varying in different cancers.

The tumor microenvironment (TME) contains various cells, with infiltrating immune cells accounting for a large proportion 8. The roles and prognostic value of infiltrating immune cells have been extensively studied in various malignant tumors 9, 10. Immunotherapy, as an alternative approach to anticancer treatment, has been developed in recent years to reactivate the adaptive and innate immune systems and create a robust antitumoral immune response. For instance, mall-molecule inhibitors of cytotoxic T lymphocyte associated antigen 4 (*CTLA4*), programmed death-1 (*PD-1*), and programmed death ligand-1 (*PD-L1*) were found to have promising antitumor effects on non-small-cell lung carcinoma and colorectal cancer 11, 12. Unfortunately, only a small proportion of patients with certain cancer types respond well to current immunotherapies 12. Thus, it is necessary to explore additional potential targets.

In this study, we evaluated the expression signature and prognostic value of the *APBB1IP* gene in pan-cancer analysis using the TCGA pan-cancer database and Kaplan-Meier Plotter. We then explored the potential relationships between *APBB1IP* expression and immune infiltration levels. Finally, the potential mechanisms were also explored by bioinformatic analysis.

## Methods

### Data Acquisition

Mutation data, RNA sequencing data, and clinical data for 33 cancer types were obtained from TCGA using UCSC Xena 13. GSE13213 14 and GSE29016 15 gene expression profiles were retrieved from the gene expression omnibus database. The inclusion and exclusion criteria adopted for the samples were (1) the loss of expression of APBB1IP; (2) the loss of follow-up information; (3) uncertain TNM stage; and (4) accompanied by other diseases. In GSE13213 and GSE29016, 117 and 68 lung cancer samples were retrieved respectively for subsequent analysis. Other data were obtained from public databases as described in the corresponding parts.

### APBB1IP gene expression analysis

The mRNA expression of APBB1IP in different cancer types was analyzed in the ONCOMINE database. 16 The threshold was set to a P-value of 0.01 and fold-change of 2. *APBB1IP* expression in normal tissue and tumor tissue was compared across cancers using the Wilcoxon test, and the results were visualized using the R packages “*ggpubr*”.

### Survival analysis

The prognosis of patients with high- tumors and low-*APBB1IP* tumors was compared using univariate Cox analysis for each cancer subtype, and genes with a *P* value < 0.05 were considered as prognostic genes. Overall survival analysis was performed via Kaplan-Meier survival analysis using the “*survival*” and “*survminer*” R packages.

### Kaplan-Meier Plotter Database Analysis

Kaplan-Meier plotter was used to assess the effects of 54,675 genes on survival using 10,461 cancer samples. These samples include 5,143 breast, 1,816 ovarian, 2,437 lung, and 1,065 gastric cancer samples on the HGU133 Plus 2.0 array with a mean follow-up of 69, 40, 49, and 33 months, respectively. The correlation between APBB1IP expression and survival in 21 different cancers was analyzed using Kaplan-Meier plotter 17. The hazard ratio (HR), 95% confidence interval and log-rank P-value were also computed.

### TIMER analysis

The TIMER database was used to systematically analyze the tumor-infiltrating immune cells (TIICs) in 32 cancer types in more than 10,000 samples from The Cancer Genome Atlas (TCGA) database 18. TIMER applies a previously published statistical deconvolution method to infer the abundance of tumor-infiltrating immune cells (TIICs) from gene expression profiles 19. Gene modules were used to analyze *APBB1IP* expression in several types of cancer and the correlation of *APBB1IP* expression with the abundance of infiltrating immune cells, after which Spearman's rho value and statistical significance were obtained. The correlation between *APBB1IP* expression and several immune cell markers was also analyzed by Spearman correlation to identify the potential subtypes of infiltrating immune cells. Immune gene markers were selected from the website of R&D Systems.

### Immune factors correlation analysis

Different immune factors lists were obtained from the Tumor Immune System Interactions Database 20, which includes immunoinhibitory and immunostimulatory factors. The Spearman method was used to determine the correlation coefficients.

### GO functional analysis and KEGG pathway enrichment analysis of DEGs

GO functional analysis is a useful method for annotating genes and identifying characteristic biological attributes from high-throughput genome or transcriptome data 21. KEGG incorporates a wide range of databases, including those on genomes, biological pathways, diseases, drugs and chemical substances 22. The Database for Annotation, Visualization and Integrated Discovery, an online bioinformatics database 23, was used for the GO functional analysis and KEGG pathway enrichment analysis, with FDR<0.05 as the cut-off criterion.

### PPI network and TF regulatory network

A PPI network was developed using the online database STRING (http://string-db.org) 24. Cytoscape software was used to construct a PPI network and analyze the interactions between APBB1IP and associated proteins 25. The iRegulon Cytoscape plugin was used to predict the TF regulatory network.

### Transcription analysis

We downloaded miRNA data and the location of potential binding sites on the 3′UTR of *APBB1IP* from miRWalk 26. The Spearman correlation between the expression of these miRNAs and *APBB1IP* was investigated using STARBASE v3.0 27.

### Genetic and epigenetic analysis

GSCALite consists of analytical modules for multi-omics data from TCGA 11 160 samples across 33 cancer types (TCGA Cancer), 746 drug data from Genomics of Drug Sensitivity in Cancer (GDSC) and the Cancer Therapeutics Response Portal (CTRP), as well as normal tissue expression data of 11 688 samples from GTEx (GTEx Normal Tissue). We used GSCALite to analyzed the single nucleotide variation, copy number variation, methylation and pathway activity 28.

### Construction of a Prognostic Model

*APBB1IP* and associated genes were evaluated by step-wise multivariate Cox regression analysis. Risk scores were calculated based on gene expression multiplied by a linear combination of a regression coefficient obtained from the multivariate Cox analysis. Patients were assigned to high- and low-risk groups according to the median risk score. The survival analysis of patients in the high- and low-risk groups was conducted using the “*survival*” R package. The receiver operating characteristic (ROC) curve was implemented by the R software package “*survival ROC*”.

## Results

### Pan-cancer Analysis of APBB1IP mRNA Expression Levels

**Figure 1:**
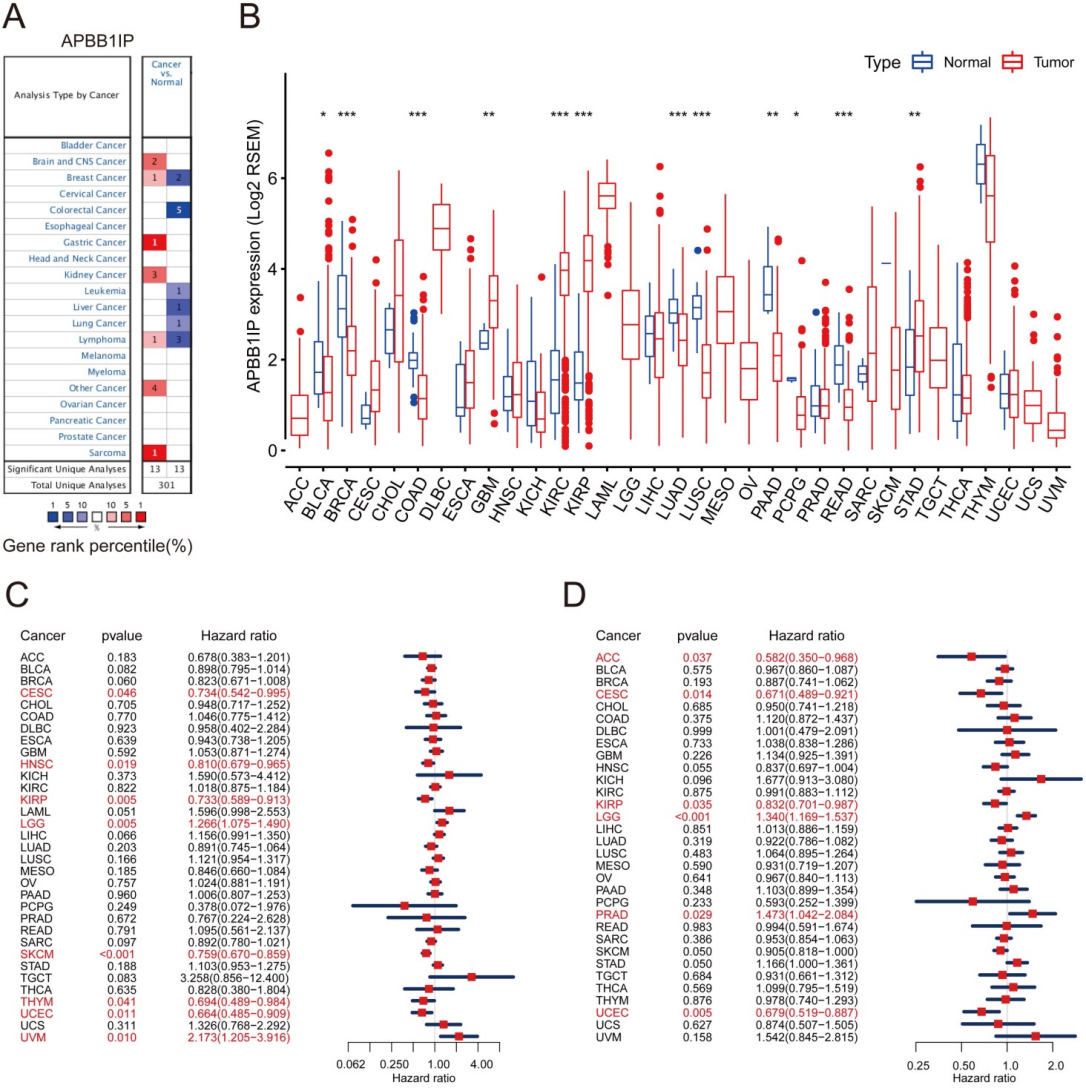
** mRNA Expression Levels Prognostic Significance of APBB1IP in different Cancers.** (A) High or low expression of APBB1IP in different human cancer tissues compared with normal tissues using the Oncomine database. The number in each cell is the amount of datasets. (B) The level of APBB1IP expression in different tumor types from the TCGA database. *P < 0.05, **P < 0.01, ***P < 0.001. (C) Correlation of APBB1IP mRNA expression with OS for different cancer types in TCGA. (D) Correlation of APBB1IP mRNA expression with RFS for different cancer types in TCGA. Red squares represent the hazard ratios. Short bars appear due to limited sample size due to which the parameters and hazard ratio could not be calculated. OS: overall survival; PFS: progression-free survival. Red Font represented *P* < 0.05.

To explore its role in cancer, the *APBB1IP* mRNA expression levels were analyzed over a cancer-wide range in Oncomine. The results revealed that the expression of *APBB1IP* was inconsistently up- or down-regulation in different cancer types (Figure 1A). The details of *APBB1IP* expression in multiple cancers are summarized in Table S1. To further evaluate *APBB1IP* expression in different cancers, we examined the expression levels of *APBB1IP* in all 33 cancer types available in TCGA the pan-cancer database (Summery of TCGA data are in Table S2). The differential expression patterns of *APBB1IP* in tumors and adjacent normal tissues are shown in Figure 1B, *APBB1IP* expression was lower in some cancers, including BLCA, BRCA, COAD, LUAD, LUSC, PAAD and READ, while others were characterized by high *APBB1IP* expression (GBM, KIRC, KIRP and STAD). These findings demonstrate the intrinsic differences in the expression of *APBB1IP* between different tumor types, and detailed analyses of *APBB1IP* expression were considered for further analysis.

### Prognostic Significance of APBB1IP Expression in Human Cancers

We next investigated the prognostic value of *APBB1IP* via pan-cancer analysis in different databases. First of all, we used univariate Cox proportional hazard regression models to analyze the association between *APBB1IP* expression with the overall survival and progression-free survival in various cancers in the TCGA. The criterion for significant association was a *P*-value of less than 0.05. As shown in the Figure 1C, *APBB1IP* was associated with poor prognosis in LGG (OS: HR = 1.266, 95% CI from 1.075 to 1.490, *p=* 0.005) and UVM (OS: HR = 2.173, 95% CI from 1.205 to 3.916, *p=* 0.010), while increased expression of APBB1IP was primarily associated with a survival advantage in patients with CESC, HNSC, KIRP, SKCM, THYM, and UCEC. Figure 1D indicated that high expression of *APBB1IP* predicted shorter RFS in patients with LGG and PRAD. In patients with ACC, CESC, KIRP and UCEC, high *APBB1IP* expression predicted better disease‐free survival. Overall- and progression-free survival curves stratified by high- and low-expression of *APBB1IP* in different types of cancer are shown in the Figure S1A and S1B, respectively.

To further examine the prognostic potential of *APBB1IP* in different cancers, the Kaplan-Meier plotter database was used to evaluate the prognostic value of *APBB1IP* based on gene chip and RNA-seq data from the GEO and EGA databases. Low *APBB1IP* expression levels were associated with poorer OS in BRCA, CESC, HNSC, KIRP, READ, SARC, THYM and UCEC. Conversely, low expression of *APBB1IP* was correlated with better OS in ESCA, LIHC, STAD and TGCT (Figure S2A-F). These results demonstrate the prognostic significance of *APBB1IP* expression in several human cancers, although their correlation may vary depending on the cancer type.

### Correlation of APBB1IP Expression with Immune Infiltration and Various Subsets of Immune Cells

**Figure 2:**
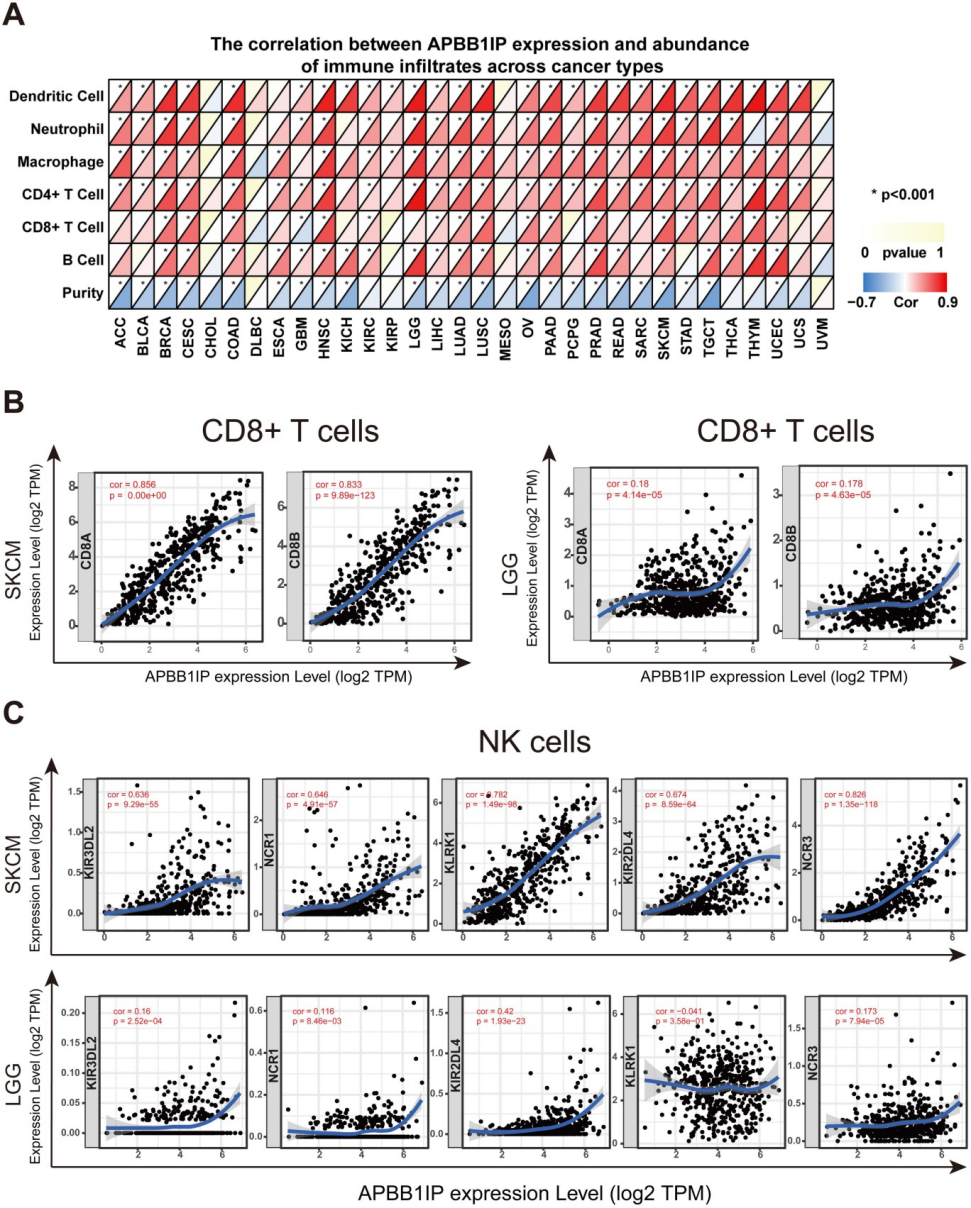
** Correlation of APBB1IP Expression with Immune Infiltration and Various Subsets of Immune Cells.** (A) The correlation between APBB1IP expression and abundance of infiltrating immune cells across cancer types. (B) Correlation of *APBB1IP* expression with markers of CD8^+^ T cells in SKCM and LGG. (C) Correlation of *APBB1IP* expression with markers of NK cells in SKCM and LGG.

It has long been recognized that lymphocytes are intimately associated with tumor cells. For instance, it was reported that the presence of TILs is associated with a more favorable prognosis in patients with breast cancer 29. Multiple correlation-based studies have implicated *APBB1IP* in signaling events critical for integrin-mediated control of immune function 4, 6, and these findings support a prognostic role of *APBB1IP* in cancer. It is likely that *APBB1IP* might influence the progression of cancer by influencing the regulation of immune infiltration. To determine the role of *APBB1IP* expression in TIL abundance, TIMER was used to obtain the Spearman correlation values for *APBB1IP* expression and the infiltration levels of various immune cells. These correlations were presented as heat maps in Figure 2A. The results revealed that *APBB1IP* expression was significantly negatively correlated with tumor purity in most cancer types, except CHOL, DLBC, KIRC, KIRP, MESO, THCA, THYM, UCS and UVM, indicating that *APBB1IP* expression in tumor tissues might be induced by the infiltrating immune cells. Higher *APBB1IP* expression in most cancers markedly increased the infiltration of immune cells, especially in BRCA, CESC, HNSC, PRAD, SKCM, TGCT and UCEC. However, In CHOL, DLBC, MESO, and UVM, the expression of *APBB1IP* showed no significant correlation with the infiltration of various immune cells.

These results demonstrate that there is a positive correlation between APBB1IP expression and immune cell infiltration levels in different cancer types, such as BRCA, CESC, HNSC, SKCM and UCEC, in which *APBB1IP* expression levels are correlated with good prognosis. Since *APBB1IP* expression is related to poor prognosis in LGG, STAD, and TGCT, we explore the clinical relevance of immune subsets using the “Survival” module in the TIMER database. Kaplan-Meier curves showed that patients with higher abundance of tumor-infiltrating B cells, CD8^+^ T cells, CD4^+^ T cells, macrophages, neutrophils and dendritic cells had shorter overall survival in LGG (Figure S2M). Similarly, in patients with STAD, high abundance of macrophages was associated with shorter survival time (Figure S2M), and the infiltration levels of macrophages and neutrophils in TGCT were correlated with shorter survival time (Figure S2M). These results indicated that *APBB1IP* might affect patient survival by interacting with tumor-infiltrating immune cells, but the anti-cancer and pro-cancer effects depend on the host environment and cancer types.

Most immune system components are implicated in the initiation and progression of melanoma 30, 31. To further determine the correlation between *APBB1IP* expression levels and various subsets of infiltrating immune cells in SKCM, we analyzed the correlation between *APBB1IP* and immune cell markers comprising subsets of T cells, B cells, monocytes, M1 and M2 macrophages, neutrophils, NK cells, and dendritic cells (DCs) in SKCM and LGG, in which *APBB1IP* expression was correlated with a poor prognosis. As shown in Table 1 and Figure 2B, after adjustments for tumor purity, *APBB1IP* expression was significantly correlated with most immune cell markers in SKCM, in addition to several markers of M1 macrophages and neutrophils. By contrast, *APBB1IP* was not significantly correlated with gene markers of CD8^+^ T cells and NK cells in LGG (Figure 2B, 2C). CD8^+^ T cells constitute the majority of TILs and directly induce cell death in tumors. Thus, a large number of CD8^+^ T cells in the tumor environment are considered to be associated with a favorable prognosis 10, 32, 33. It is also well known that NK cells have spontaneous killing activity against tumor cells 34. These findings could partly explain why *APBB1IP* is related to a bad prognosis in LGG.

### Immune Factors and Functional Analysis

**Figure 3:**
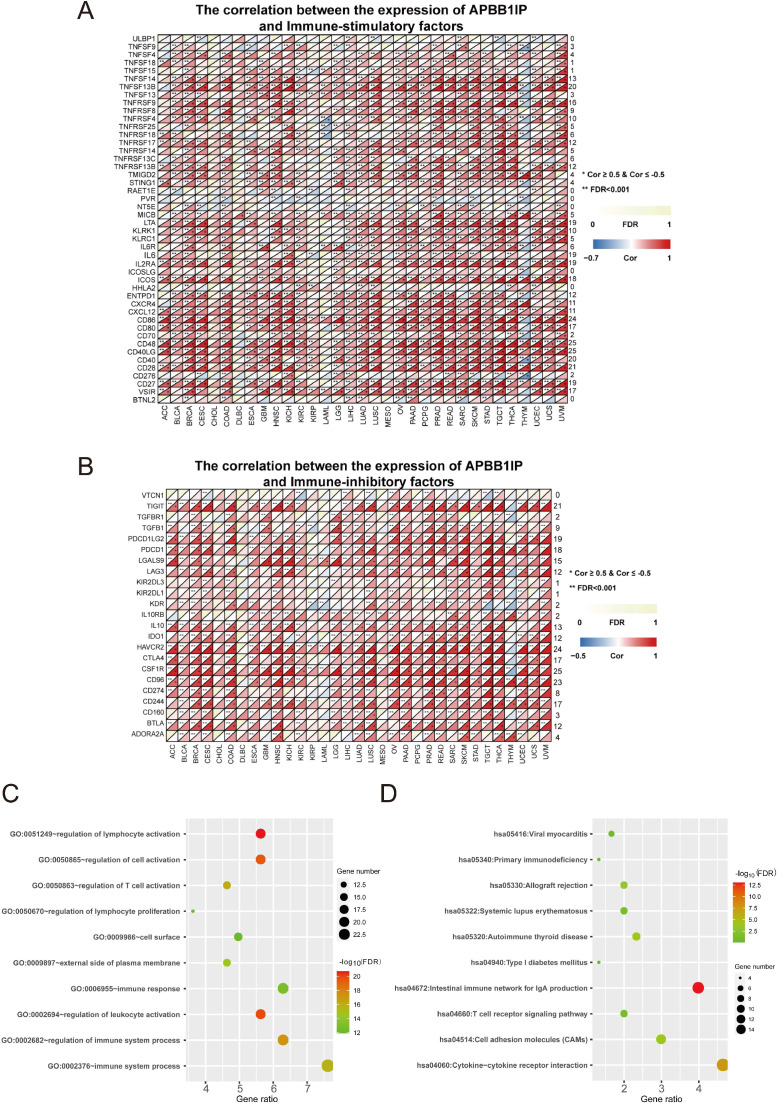
** Immune Factors and Functional Analysis.** (A) The correlation between the expression of APBB1IP and immunostimulatory factors. (B) The correlation between the expression of APBB1IP and immunoinhibitory factors. (C) GO analysis of significantly correlated immune factors. (D) KEGG pathway analysis of correlated immune factors.

To clarify the mechanisms underlying the involvement of *APBB1IP* in the enhancement of immune-cell infiltration, we calculated the Spearman correlations of *APBB1IP* expression with immune factors in the TISIDB database, including immunoinhibitory and immunostimulatory factors. These results are presented as heat maps in Figure 3A, and 3B. The results indicated that *APBB1IP* expression is positively correlated with both immunoinhibitory and immunostimulatory factors in the majority of cancers. We chose a correlation coefficient > 0.5 and FDR> 0.001 as cut-off criteria. According to this analysis, *APBB1IP* was not significantly correlated with majority immune factors in CHOL, DLBC, KIRP, LAML, MESO, PCPG and THYM. Especially in the latter, most immune factors showed a negative correlation with *APBB1IP*, which was opposite to other cancer types, suggesting that the immune microenvironment of THYM is different from other cancers. Accordingly the mechanism by which APBB1IP affects the prognosis of THYM might be different.

Next, 35 genes (Table S3) that were significantly associated with *APBB1IP* expression according to the cut-off standard in greater than or equal to 15 cancers, were selected as candidate genes for GO and KEGG pathway analysis in DAVID (https://david.ncifcrf.gov/summary.jsp). Figure 3C shows the top 10 most highly enriched GO items. Specifically, the immune factors were mainly enriched in biological processes (BPs) related to the regulation of lymphocyte activation, regulation of cell activation, regulation of T cell activation, regulation of lymphocyte proliferation, immune response, regulation of leukocyte activation, regulation of immune system processes, and immune system processes. In terms of function, the immune factors were predicted to be associated with the cell surface and the external side of plasma membrane. More specific information is listed in Table S4. Additionally, the most significantly enriched KEGG pathways are displayed in Figure 3D. The immune factors were enriched in the categories of viral myocarditis, primary immunodeficiency, allograft rejection, systemic lupus erythematosus, autoimmune thyroid disease, type I diabetes mellitus, intestinal immune network for IgA production, T cell receptor signaling pathway, cell adhesion molecules (CAMs) and cytokine-cytokine receptor interaction. The detailed information is shown in Table S5.

### PPI Network Establishment and Transcription and Epigenetics Analysis of APBB1IP in Pan-cancer analysis

**Figure 4:**
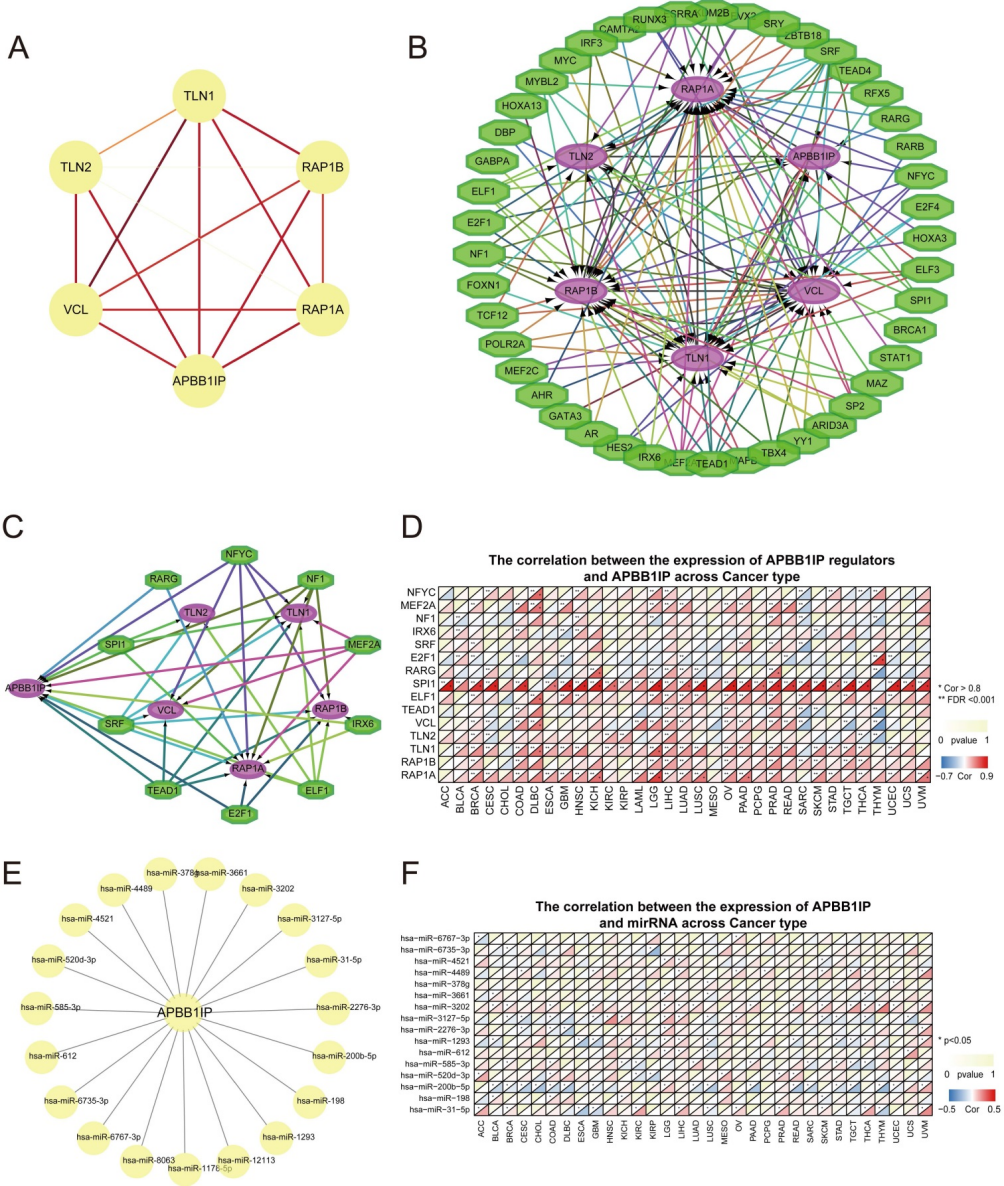
** Establishment of a PPI Network and Pan-cancer Analysis of Transcription and Epigenetic Factors of APBB1IP.** (A) PPI network of APBB1IP and its interacting proteins. The PPI consists of 6 nodes and 15 edges, with an average node degree of 5 and average local clustering coefficient of 1 at a PPI enrichment p-value of 3.69 × 10^-4^. The color and thickness of the solid line represents the strength of the relationship. (B) TF-target regulatory network of APBB1IP and its interacting proteins. Green polygons represent TFs, the purple ellipses represent the target proteins. (C) The TFs that target APBB1IP. Green polygons represents TFs, the purple ellipses represent the target proteins. (D) Correlation between the expression of APBB1IP regulators and APBB1IP across cancer types. (E) MiRNAs that target APBB1IP. (F) The correlation between the expression of APBB1IP and miRNAs across cancer types.

Protein-protein interaction (PPI) analysis can reflect the molecular mechanisms of physiological and pathological changes that drive cancer progression. The PPI network of APBB1IP and its protein partners was constructed using the STRING database (https://string-db.org/) and Cytoscape software (Figure 4A). The network contained 6 nodes and 15 edges, with the 5 predicted proteins interacting with APBB1IP including members of the *RAS* oncogene family (*RAP1A* and *RAP1B*), *talin 1* (*TLN1*), *talin 2* (TLN2), and *vinculin* (*VCL*). These genes were further considered for downstream analysis of the *APBB1IP* network. Subsequently, a TF regulatory network with 45 nodes was predicted using the iRegulon Cytoscape plugin (Figure 4B). Among these TFs, *NFYC*, *MEF2A*, *NF1*, *IRX6*, *SRF*, *E2F1*, *RARG*, *SPI1*, *ELF1* and *TEAD1* were identified as targeting *APBB1IP*. Additionally, they were also predicted to regulate the interacting proteins (Figure 4C). Subsequently, we explored the Spearman correlations of *APBB1IP* and these transcriptional regulators together with the protein partners of *APBB1IP*, as shown in Figure 4D. Among the transcriptional regulators, the expression of *SPI1* was significantly positively associated with *APBB1IP* in the majority of cancers. Furthermore, it was found to target *TLN1*, *TLN2* and *RAP1A* as well. *SPI1/PU.1* is a member of the ETS family that is critical for specifying cell fate and proper hematopoietic differentiation 35. *SPI1* plays a crucial part in the self-renewal of hematopoietic stem cells (HSCs) as well as in myeloid and B lymphoid differentiation 36, 37. Based on these findings, *SPI1* might function by regulating the transcription of *APBB1IP* and other associated proteins. Subsequently, using miRWalk (including miRBase, TargetScan, miRDB, and miRTarBase), we predicted 19 miRNAs that could potentially target the 3′UTR of *APBB1IP* (Figure 4E). Using STARBASE v3.0 with *p* < 0.05 as screening criteria, we investigated the Spearman correlation between miRNAs and the expression of *APBB1IP* in 32 kinds of cancers from TCGA (Figure 4F). The hsa-miR-200b-5p was predicted to have a significant inhibitory effect on *APBB1IP* in BLCA, BRCA, CESC, CHOL, COAD, DLBC, GBM, LUAD, LUSC, PAAD, READ, SARC, STAD, TGCT and THYM.

### Analysis of Genetic Mutations and Methylation of APBB1IP

**Figure 5:**
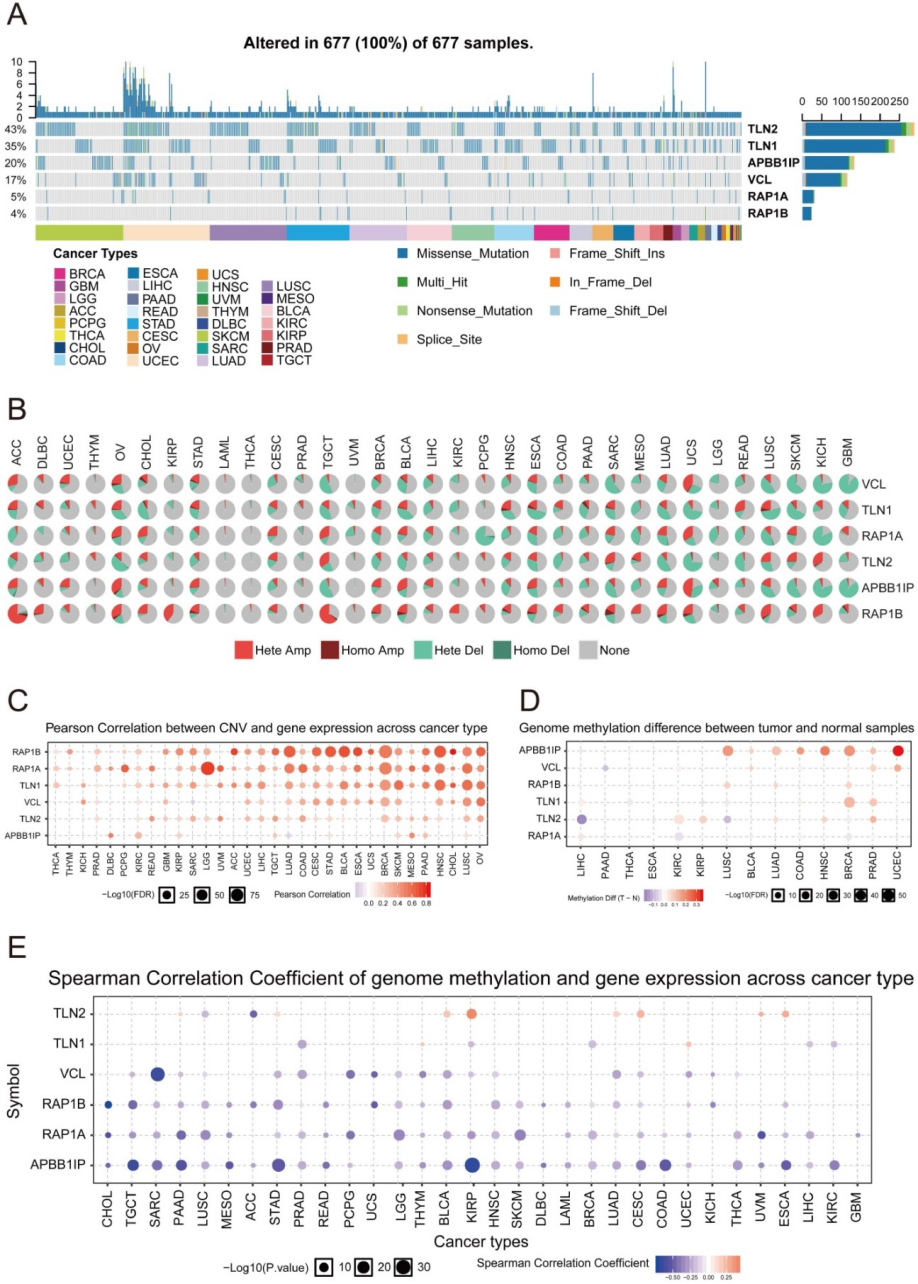
** Genetic Mutation and Methylation Analysis of APBB1IP.** (A) SNV frequency of APBB1IP and its protein partners. Each gray vertical bar represents a patient. The side and top column diagrams show the numbers of variants in each sample or each gene. (B) Pan-cancer analysis of heterozygous/homozygous CNV of APBB1IP and its protein partners. Hete Amp: heterozygous amplification; Hete Del: heterozygous deletion; Homo Amp: homozygous amplification; Homo Del: homozygous deletion; None: no CNV. (C) Pan-cancer analysis of the CNV correlation with mRNA of APBB1IP and its protein partners. Genes whose mRNA expression significantly correlates with CNV percentage (FDR<=0.05) are shown in the figure. Blue bubbles represent a negative correlation, and red bubbles represent positive correlation (genes having a high frequency of CNV are shown in a deeper color, indicating a higher correlation. The size of each bubble represents statistical significance. (D) Bubble map of the differential methylation of APBB1IP and its protein partner s between normal and cancer samples in TCGA. The significance of differences was analyzed using Student's *t*-test. Blue dots represent down-regulation of methylation in tumors, and red dots represent up-regulation of methylation in tumors; the darker the color, the greater the difference. The size of the bubble represents statistical significance. (E) Correlation between methylation, APBB1IP and its protein partners in cancer samples from TCGA. The data were subjected to Person correlation analysis. Blue bubbles indicate that the gene methylation level is up-regulated and gene expression is down-regulated. Red bubbles indicate that the gene methylation level and gene expression are up-regulated. The darker the color, the higher the correlation. The size of the bubble represents the statistical significance.

To further understand how *APBB1IP* expression is altered across different cancers, we explored the Single Nucleotide Variation (SNV) profile of *APBB1IP* and its protein partners using GSCALite 28 (Figure 5A). All of the 677 analyzed tumors showed at least 1 mutation. TLN1 had the highest SNV frequency (43%) among the analyzed tumors, followed by TLN2 (35%). For *APBB1IP*, the SNV frequency was 20%, at the cancer level. Additionally *APBB1IP*-associated SKCM exhibited the highest number of mutations (32), followed by LUSC (22) and UCEC (21). The most frequent DNA alterations of these 6 genes in the pan-cancer analysis were missense mutations. CNVs were reported as frequent pathogenic events in cancers, which could contribute to increased DNA instability and occurrence of genomic imbalance 38. Figure 5B shows the heterozygous/homozygous CNV status of each gene in each cancer. Next, the Pearson correlation was between gene expression and CNV was analyzed in different cancers to identify the genes significantly affected by CNV. As shown in Figure 5C, the expression of *TLN1* and *TLN2* was positively associated with CNV in most cancers, except for *APBB1IP*, *RAP1B*, and *RAP1A*.

Since DNA methylation is involved in gene regulation and cell differentiation 39, we next explored whether methylation is involved in the regulation of *APBB1IP*. We found that the methylation of *APBB1IP* was significantly up-regulated in LUSC, BLCA, COAD, HNSC, BRCA, PAAD, and UCEC, while co-methylation patterns of *APBB1IP* and the protein partners of APBB1IP were not observed (Figure 5D). In addition, the expression of *APBB1IP* and protein partners was mainly negatively correlated with methylation, with only a few positive correlations (Figure 5E), providing clues for the deregulation of *APBB1IP*.

### Cox Progression Analysis and Identification of a Prognostic Signature in Lung Cancer

**Figure 6:**
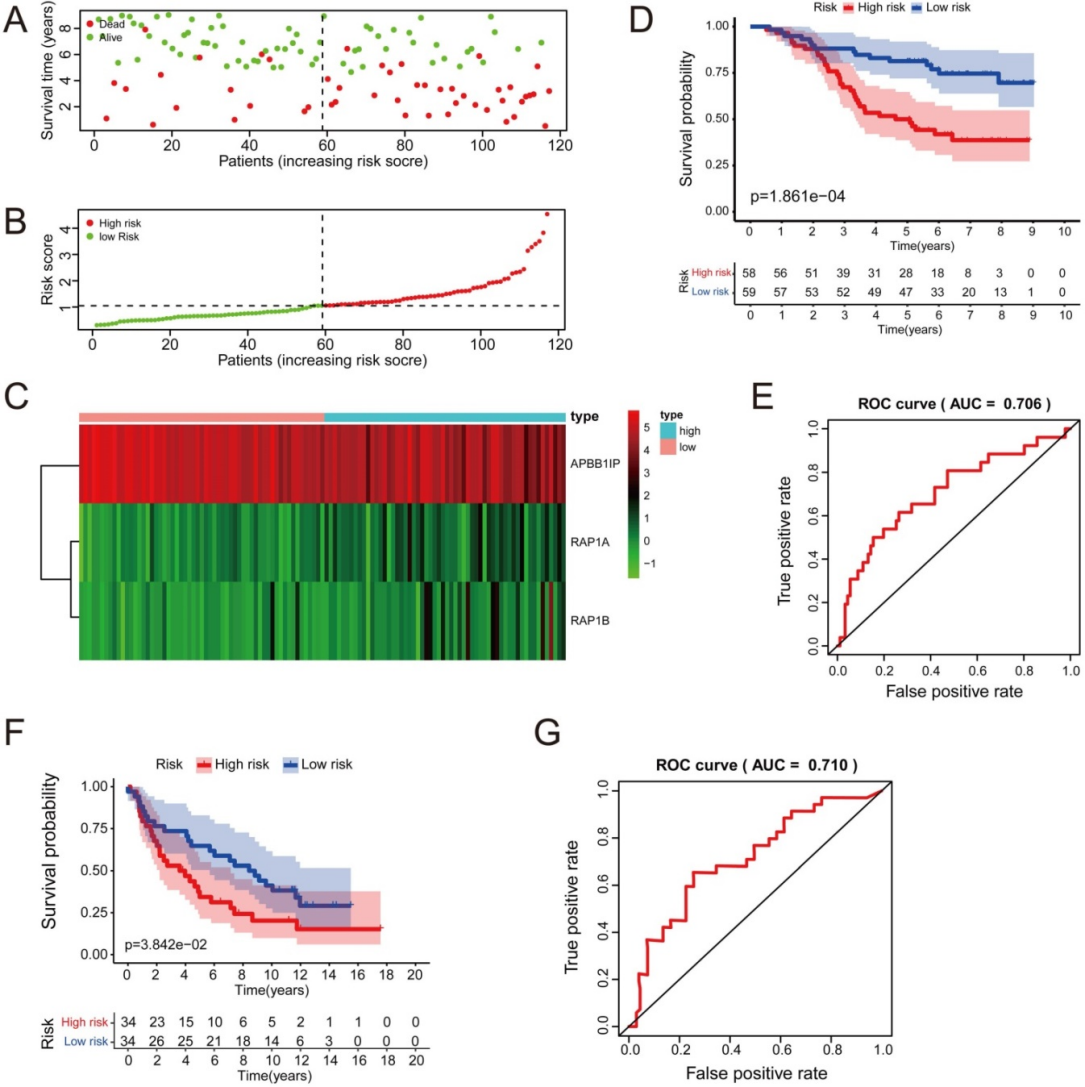
** Cox Regression Analysis and Identification of a Prognostic Signature in Lung Cancer.** (A) The risk score distribution of HCC patients in the GSE13213 dataset. (B) Patients' survival status distribution. (C) A heatmap of APBB1IP and RAP1B in low- and high-risk groups. (D) The survival curves of GSE13213 patients in low- and high-risk groups. (E) Receiver operating characteristic curve (ROC) analysis predicting overall survival using the risk score in GSE13213. (F) The survival curves of GSE29016 patients in low- and high-risk groups. (G) Receiver operating characteristic curve (ROC) analysis predicting overall survival using the risk score in GSE29016.

**Figure 7:**
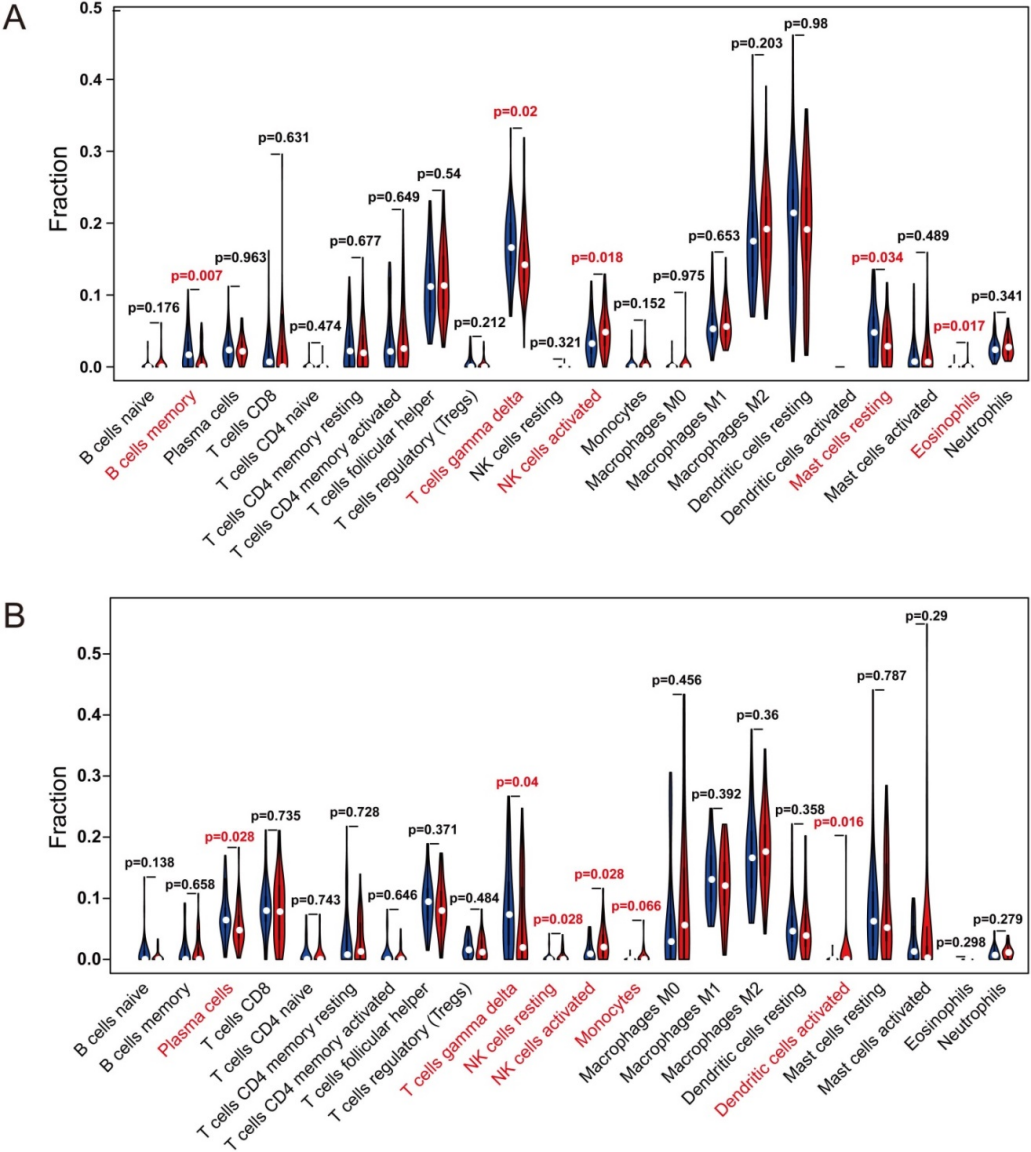
The Relationships Between the Risk Score Model and Immune Cell Infiltration. (A, B) Violin plot showing the relationship between the risk score with the abundances of different types of infiltrating immune cells. Red color represents the high-risk group while blue color represents the low-risk group. Differential abundance of immune cell types was observed between the high and low-risk groups. (A) GSE13213. (B) GSE29016.

Multivariate Cox analysis was performed for *APBB1IP*, *RAP1A*, *RAP1B*,* TLN1*, *TLN2* and *VCL* in GSE13213. Then, *APBB1IP*, *RAP1B* and *RAP1B* were finally selected to establish a prognostic model. The model was described using the formula *risk score = (-0.85392 × expression level of APBB1IP)+(0.422303 × expression level of RAP1A) +(0.377711 × expression level of RAP1B).* All three genes were prognostic for increased risk*,* including *APBB1IP* (HR = 0.43, 95% CI = 0.25 to 0.73, *p* value= 0.002), *RAP1A* (HR = 1.53, 95% CI = 1.00 to 2.33, *p* value= 0.049)*,* and *RAP1B* (HR = 1.46, 95% CI = 1.08 to 1.96, *p* value= 0.013). Multivariate Cox regression was used to calculate regression coefficients. Risk scores were based on gene expression levels multiplied by the corresponding regression coefficients. Subsequently, 117 lung cancer samples were divided into a high-risk group (*n = 59*) and a low-risk group (*n = 58*) based on the median risk score (Figure 6B). The survival status and survival time in the model group are shown in Figure 6A, while Figure 6C shows a gene expression heatmap of the low- and high-risk groups. Survival analysis indicated that patients in the high-risk group showed markedly poorer overall survival than those in the low-risk group (*p=1.861e-04*; Figure 6D). According to the ROC curve of 5-year OS (Figure 6E), an area under curve (AUC) value of 0.706 (*>0.7*), indicating that this prognostic model exhibited good sensitivity and specificity. To identify the relationship between risk score model and clinicopathological characteristics in lung cancer patients, we further analyzed the risk score level in lung cancer patients at different clinical stages. As revealed in Table 2, risk score based on this prognostic model was significantly associated with T stage, N stage, TNM stage and relapse (both *P* < 0.05). Subsequently, the GSE29016 dataset was used as the testing cohort. The Kaplan-Meier analysis shown in Figure 6F indicated that the high-risk group had poorer overall survival (*p=3.842e-02*). Time-dependent ROC curves showed that the model had good accuracy with a value of 0.710 in 5 years (Figure 6G). Moreover, analysis of the correlation of risk stratification with clinicopathological data for patients with lung cancer also showed that TNM stage was significant associated with risk stratification in the testing cohort (Table S6). Next, the relationships between the risk score model and immune cell infiltration was investigated. As shown in Figure 7A, NK cells activation and eosinophil infiltration were positively correlated with the risk score. However, negative correlations were observed between the risk score and memory B cells, gamma delta T cells and resting mast cells. Similarly, the high- and low-risk groups also showed differential immune cell abundance in GSE29106 (Figure 7B).

## Discussion

APBB1IP contains Ras association (RA) and pleckstrin homology (PH) domains and proline-rich regions, which are defining features of the *Mig-10/RIAM/Lamellipodin* (MRL) family of adapter proteins. It was identified as a Rap1-binding protein important for integrin-mediated migration and activation of leukocytes 2. In spite of the important roles of *APBB1IP* in the immune system, *APBB1IP* has not been well-studied in immuno-oncology. Here, we conducted a pan-cancer analysis of the expression profile and prognostic significance of *APBB1IP* and revealed its potential role in tumor immunology via bioinformatics analysis.

In this study, the expression levels of *APBB1IP* were examined and the prognostic landscape in pan-cancer analysis were visualized using independent datasets in Oncomine and Kaplan-Meier plotter, as well as TCGA data for 33 types of cancer. According to the combined results from the Oncomine and TCGA data, *APBB1IP* was highly expressed in KIRC, KIRP, STAD, sarcoma, as well as brain and CNS cancers compared to corresponding normal tissues, while it had lower expression levels in BLCA, BRCA, COAD, LUAD, LUSC, PAAD, PCCG, READ, liver cancer and leukemia. From the comprehensive TCGA and Kaplan-Meier plotter data, we found consistent prognostic correlations of *APBB1IP*. Specifically, decreased *APBB1IP* expression was correlated with poor prognosis in most tumor types (CESC, HNSC, KIRP, THYM, UCEC). However, STAD was an exception where high levels of *APBB1IP* expression indicated a poorer prognosis. The discrepancies in *APBB1IP* levels and prognosis in different cancer types in different databases might be a reflection of different data collection approaches and underlying mechanisms pertinent to different biological properties. Notably, *APBB1IP* was up‐regulated in KIRP, but high *APBB1IP* expression indicated a better prognosis in this cancer. This counterintuitive finding can be explained by the small sample size of the normal group (*n = 32*), which might have led to unreliable data on the expression of *APBB1IP* in KIRP. Hence, in future studies, researchers need to collect more samples to verify the expression of *APBB1IP* in KIRP. In addition, *APBB1IP* might play distinct roles in the initiation and progression of KIRP. To confirm this, further studies are needed to explore the expression of *APBB1IP* in tumors with different stages or grades and analyze the relationship between *APBB1IP* expression and survival of KIRP patients with different tumor stages or grades.

Another important finding of this study is that *APBB1IP* expression is correlated with diverse immune-cell infiltration levels in most cancer types (Figure 2A). Moreover, the correlation between *APBB1IP* and the expression of immune regulators indicate a role of *APBB1IP* in regulating tumor immunology in different cancers (Figure 3A). Interestingly, although the prognostic implications of *APBB1IP* were not the same in different cancer types, *APBB1IP* expression was consistently positively correlated with the immune-cell infiltration levels in these cancers. Accordingly, tumor infiltration by the same immune cells may have a different effect on the prognosis in different cancer types. For example, high infiltration of B cells, CD8^+^ T cells, neutrophils and dendritic cells is associated with poor prognosis in LGG, but also with better prognosis in SKCM (Figure S2M). Furthermore, multiple studies have reported differences in the correlation between intratumoral immune-cell activity and survival across different cancer types 40-44. Notably, the strongest positive correlation was observed between the expression of specific markers for CD8^+^ T cells and NK cells and *APBB1IP* expression in SKCM; while the correlation in LGG was lower (Figure 2B-C). CD8^+^ T cells and NK cells are well known as effector cells in the tumor microenvironment via their cytolytic activity 45-48. This might be another reason for the different prognostic implications of *APBB1IP* in SKCM and LGG.

*RAP1A*, *RAP1B*, *TLN1*, *TLN2*, and *VCL* were predicted to interact with *APBB1IP*. *Talin1* and* talin2* are large (270 kDa) cytoplasmic adapter proteins (Figure 4A) 49, 50. In resting cells, a large portion of *talin* proteins resides in the cytoplasm in a closed, auto-inhibited conformation 51, 52. Following stimulation, *talin* is efficiently recruited to the plasma membrane and transformed from its auto-inhibitory conformation to trigger integrin activation. *Talin*-mediated integrin activation is highly dependent on the membrane-anchored small *GTPase Rap1* protein family 53, 54. It was suggested that *talin* membrane recruitment is triggered through APBB1IP, which binds the *talin* rod domain and thereby links it to the plasma membrane 55, 56. This *Rap1-RIAM-talin* pathway is crucial for leukocyte β2 integrin activation 6, 7. *Vinculin* (*VCL*) is a key adaptor molecule that links adhesion complexes to actin filaments in integrin-based cell extracellular matrix (ECM) adhesions or cell-cell junctions 57. *APBB1IP* was reported to promote the migration and invasion of melanoma cells 7, and we speculated that *APBB1IP* might interact with *VCL* to achieve this function. According to our results, high *APBB1IP* expression indicated poor prognosis in SKCM, and we deduced that the promoting effect of *APBB1IP* on the migration ability of SKCM might not be associated with its prognosis, while the influence on immune infiltration might play a leading role.

In this study, we found that *RAP1A/B* and *TLN1* expression is positively associated with *APBB1IP* (Figure 4D). Thus, we displayed the TFs which might target *APBB1IP* and its interacting partners (Figure 4B). We observed that TFs targeting *APBB1IP* could target its interacting partners at the same time (Figure 4C). In addition to for TFs, methylation and miRNAs can also regulate the protein expression levels. According to our results, the methylation levels of *APBB1IP* and *RAP1A/B* were negatively correlated with gene expression (Figure 5E). More specifically, *APBB1IP* methylation was higher in LUSC, BLCA, LUAD, COAD, BRCA, HNSC, PRAD, and UCEC tumor tissues than in normal control tissues (Figure 5D). In addition, miR-200b-5p was identified as possibly targeting APBB1IP and it was negatively associated with the expression of *APBB1IP* in most cancers.

Nevertheless, although we integrated information across multiple databases, this study still has some limitations. Since large-scale microarray and sequencing data were initially collected by analyzing tumor tissue information, the cell-level analysis of immune cell markers could have introduced systematic bias. To overcome this problem, future studies relying on methods with a higher resolution, such as single-cell RNA sequencing, should be performed 58, 59. Next, we could not prove that *APBB1IP* affected patient survival through immune infiltration even though we found that *APBB1IP* expression was correlated with both immune cell infiltration and patient survival in cancers. Hence, future prospective studies are needed to explore the relationship between *APBB1IP* expression and immune infiltration in a cancer patient population. Finally, we only conducted a bioinformatic analysis of *APBB1IP* expression and patient survival across different databases, and further mechanistic studies on *APBB1IP* at the cellular and molecular levels could help clarify its functions in cancer initiation and progression.

## Supplementary Material

Supplementary figures and tables.

## Figures and Tables

**Table 1:** Correlation between APBB1IP and markers of various immune cells in the Tumor Immune Estimation Resource (TIMER)

Description	Gene Markers	SKCM				LGG			
		None		Purity		None		Purity	
		Cor	*P*	Cor	*P*	Cor	*P*	Cor	*P*
CD8^+^ T cell	CD8A	0.8560732	***	0.7694524	***	0.1797299	***	0.0526211	0.2508652
	CD8B	0.8331593	***	0.7260344	***	0.1783245	***	0.0817846	0.0740368
T cell (general)	CD3D	0.8765923	***	0.7843417	***	0.3926254	***	0.341427	***
	CD3E	0.8909953	***	0.81022	***	0.417972	***	0.3857384	***
	CD2	0.8975331	***	0.8229912	***	0.4173085	***	0.3923904	***
B cell	CD19	0.6846681	***	0.5602065	***	0.3834718	***	0.3352988	***
	CD79A	0.724277	***	0.5894023	***	0.324637	***	0.3542463	***
Monocyte	CD86	0.9296171	***	0.8931612	***	0.9074751	***	0.8943717	***
	CD115 (CSF1R)	0.905323	***	0.867535	***	0.9069494	***	0.8913472	***
TAM	CCL2	0.6663165	***	0.5284483	***	0.4837392	***	0.4375916	***
	CD68	0.5852682	***	0.450489	***	0.829085	***	0.8148534	***
	IL10	0.716315	***	0.614492	***	0.6101679	***	0.5730547	***
M1 Macrophage	INOS (NOS2)	0.0510847	0.268534	0.0559468	0.232609	-0.1511358	***	-0.1781116	***
	IRF5	0.7086537	***	0.5635679	***	0.8953138	***	0.8761291	***
	COX2 (PTGS2)	0.0642153	0.164114	-0.0220597	0.638106	0.1411687	**	0.070951	0.1213558
M2 Macrophage	CD163	0.7727474	***	0.7078413	***	0.3657231	***	0.3589378	***
	VSIG4	0.7660752	***	0.7124946	***	0.7985103	***	0.7744972	***
	MS4A4A	0.8297304	***	0.7714602	***	0.5797796	***	0.5803033	***
Neutrophils	CD66b (CEACAM8)	-0.0298919	0.5175338	0.0014881	0.9746913	0.0356518	0.4190064	0.0322264	0.4821109
	CD11b (ITGAM)	0.7985079	***	0.7504803	***	0.9015692	***	0.8829104	***
	CCR7	0.7806993	***	0.6267265	***	0.2095446	***	0.1908896	***
Natural killer cell	KIR2DL1	0.3978069	***	0.2549098	***	0.018379	0.6770348	0.0617757	0.1775405
	KIR2DL3	0.5712978	***	0.4038145	***	0.1169197	**	0.1191511	**
	KIR2DL4	0.6744853	***	0.5261334	***	0.4197599	***	0.4394195	***
	KIR3DL1	0.53491	***	0.3765632	***	-0.0278331	0.5281487	-0.0256187	0.5763447
	KIR3DL2	0.6360165	***	0.4703593	***	0.160454	***	0.1668523	***
	KIR3DL3	0.1615421	***	0.0930881	***	0.0763603	0.0831139	0.0825424	0.071389
	KIR2DS4	0.4411108	***	0.3118604	***	0.1341571	**	0.1188675	**
	KLRK1 (NKG2D)	0.7824953	***	0.6765392	***	-0.0405603	0.357716	0.0365504	0.4252862
	NCR1 (NKp46)	0.6462555	***	0.5564235	***	0.1158136	**	0.1606459	***
	NCR2 (NKp44)	0.3058505	***	0.2514162	***	0.0304199	0.490514	0.0647637	0.1574441
	NCR3 (NKp30)	0.8255278	***	0.7169654	***	0.172838	***	0.1768626	***
Dendritic cell	HLA-DPB1	0.868107	***	0.7793636	***	0.6505592	***	0.6235519	***
	HLA-DQB1	0.8030186	***	0.6833435	***	0.4962863	***	0.4617414	***
	HLA-DRA	0.8805606	***	0.8017141	***	0.7050379	***	0.6814707	***
	HLA-DPA1	0.8493377	***	0.7661858	***	0.6513003	***	0.6270917	***
	BDCA1 (CD1C)	0.6545897	***	0.4916537	***	0.3206443	***	0.3254187	***
	BDCA4 (NRP1)	0.4935402	***	0.4514923	***	0.1058552	*	0.1603316	***
	CD11c (ITGAX)	0.6902283	***	0.5453348	***	0.7899023	***	0.7551692	***
Th1	T-bet (TBX21)	0.8701187	***	0.7846811	***	0.2129897	***	0.2334805	***
	STAT4	0.7799773	***	0.671695	***	-0.1770701	***	-0.2653241	***
	STAT1	0.643486	***	0.5645606	***	0.3297585	***	0.3193543	***
	IFNγ (IFNG)	0.7535284	***	0.6265012	***	0.2479673	***	0.2208263	***
	TNFα (TNF)	0.6784416	***	0.5088296	***	0.3810268	***	0.3364843	***
Th2	GATA3	0.7460884	***	0.5580142	***	0.3701424	***	0.3215647	***
	STAT6	0.064225	0.1640502	0.120339	*	0.5565766	***	0.4636034	***
	STAT5A	0.2495179	***	0.3128001	***	0.7509945	***	0.7019067	***
	IL13	0.2016526	***	0.1185491	*	-0.0110191	0.8028152	-0.0236849	0.6054741
Tfh	BCL6	0.3600216	***	0.3065975	***	0.203324	***	0.2535651	***
	IL21	0.5664735	***	0.4559844	***	0.1068566	*	0.098485	*
Th17	STAT3	0.3361749	***	0.3549276	***	0.4724055	***	0.5021622	***
	IL17A	-0.0944109	*	-0.191403	***	-0.0189334	0.6678661	-0.0356259	0.4370972
Treg	FOXP3	0.7678603	***	0.6220553	***	-0.1794219	***	-0.1521192	***
	CCR8	0.7853676	***	0.6941604	***	0.1045328	*	0.0986246	*
	STAT5B	0.3224104	***	0.4384412	***	-0.0256525	0.5609735	0.0785814	0.0861259
	TGF *β (TGFB1)*	0.5557131	***	0.447552	***	0.8019722	***	0.7831079	***
T cell exhaustion	PD1 (PDCD1)	0.8308004	***	0.7229568	***	0.3971191	***	0.3693027	***
	CTLA4	0.557273	***	0.390242	***	0.3747752	***	0.3282445	***
	LAG3	0.8049163	***	0.6924997	***	0.1894906	***	0.2319562	***
	TIM3 (HAVCR2)	0.9274436	***	0.8854098	***	0.9365782	***	0.9274279	***

**Table 2:** Association between the clinicopathologic parameters and the risk score levels in GSE13213

Clinical variables	Total	Risk score levels		*p* value
		Low	High	
**Gender**				0.228
Male	60	27	33	
Female	57	32	25	
**Age (years)**				0.779
≤60				
>60				
**Smoking**				0.164
NO	56	32	24	
YES	61	27	34	
**T Stage**				
T1+T2	104	56	48	**0.036**
T3+T4	13	3	10	
**N Stage**				**0.039**
N0	87	48	39	
>N0	30	10	20	
**TNM Stage**				**0.048**
I+II	92	50	42	
III+IV	25	8	17	
**Relapse**				**0.002**
NO	59	38	21	
YES	58	21	37	
**EGFR status**				0.619
WT	72	35	37	
MUT	45	24	21	
**KRAS status**				0.755
WT	102	52	50	
MUT	15	7	8	
