# Supplementary material for: Immunological Role and Prognostic Value of APBB1IP in Pan-Cancer Analysis
Source: J Cancer. 2021 Jan 1;12(2):595–610. doi: 10.7150/jca.50785 (PMC7738982; doi:10.7150/jca.50785)
Supplement: Supplementary file 1 — Supplementary figures and tables. [file jcav12p0595s1.pdf]

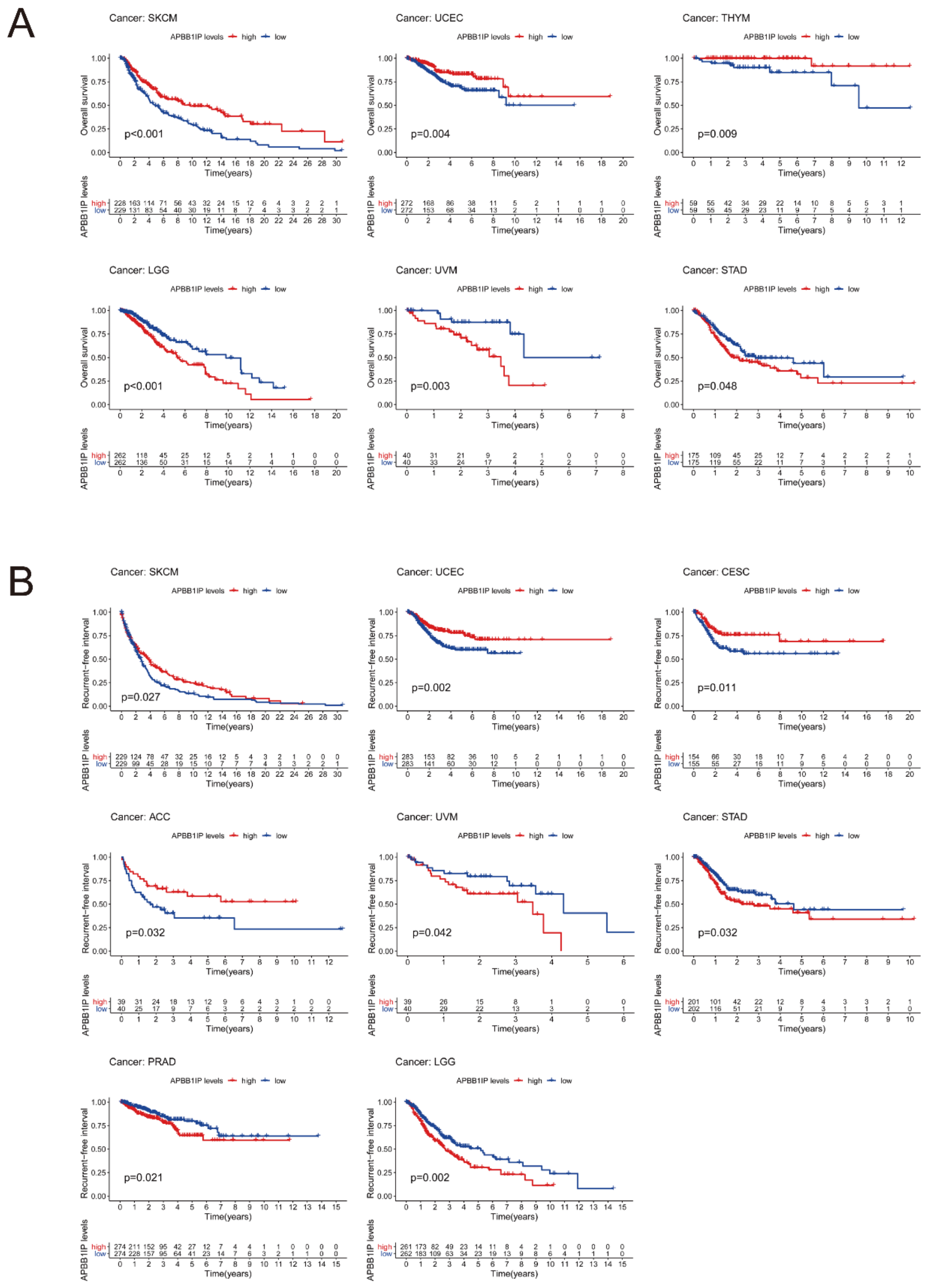

Figure S1. Correlation of APBB1IP expression with OS and RFS in diverse types of cancer in TCGA. (A) SKCM; UCEC; THYM; LGG; UVM; STAD. (B) SKCM; UCEC; THYM; LGG; UVM; STAD.

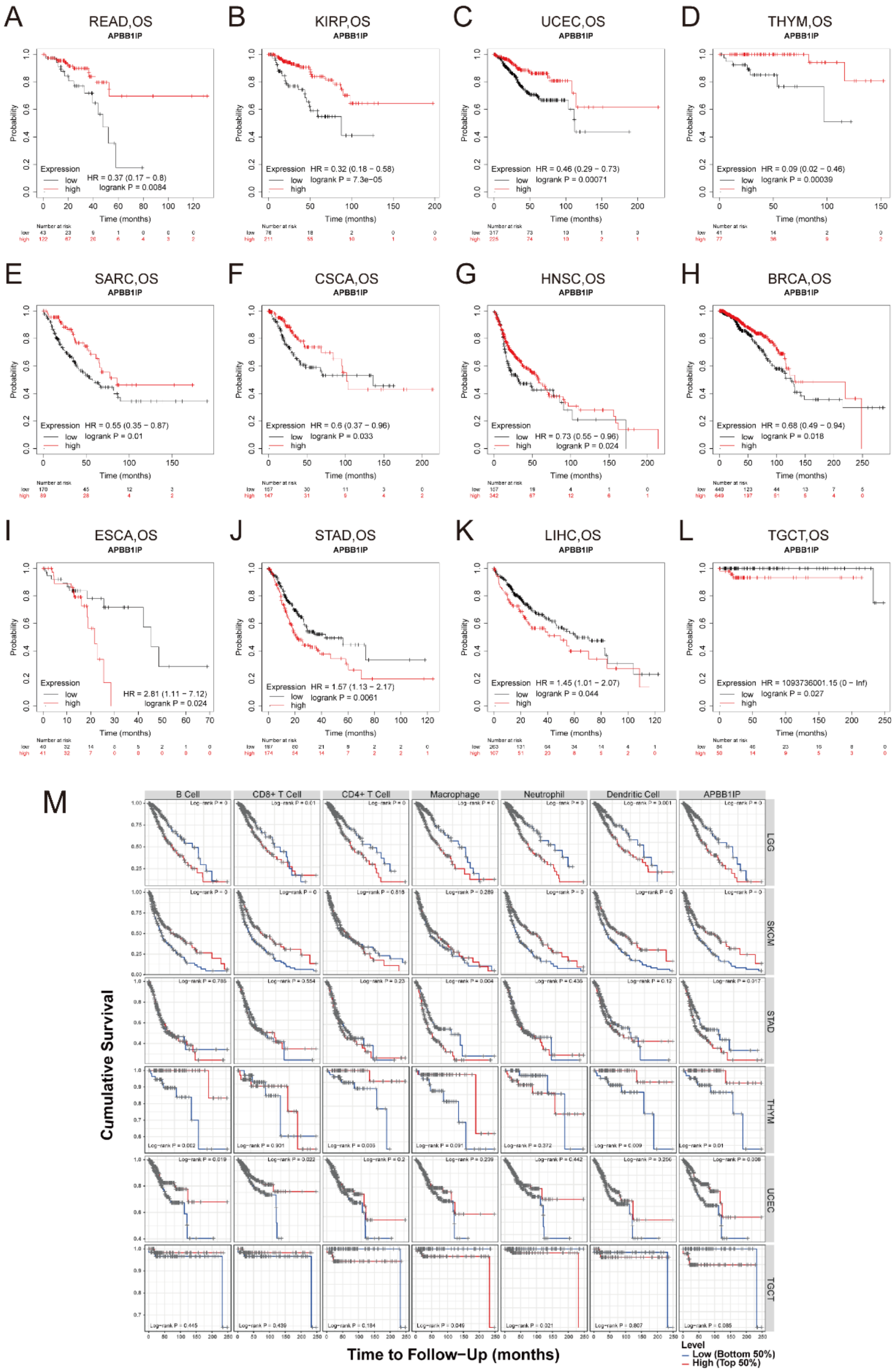

Figure S2. (A-L) Correlation of APBB1IP expression with OS in diverse types of cancer in the Kaplan-Meier plotter database, (A) READ, (B) KIRP, (C) UCEC, (D) THYM, (E) SARC, (F)

CSCA, (G) HNSC, (H) BRCA, (I) ESCA, (J) STAD, (K) LIHC, (L) TGCT. (M) Kaplan-Meier plots for infiltrating immune cells and the survival differences in different cancer types.

Table S1. APBB1IP expression in cancerous versus normal tissue in ONCOMINE.

Cancer Site	Cancer Type	P Value	Fold Change	Sample Size	Reference
Brain and CNS Cancer	Diffuse Astrocytoma	0.00048	2.11	30	Sun Brain
	Glioblastoma	0.003	6.549	25	Lee Brain
Breast Cancer	Invasive Breast Carcinoma Stroma	7.7E-13	6.657	59	Final Breast
	Breast Phyllodes Tumor	4.4E-05	-2.417	149	Curtis Breast
	Mucinous Breast Carcinoma	0.001	-3.556	65	TCGA Breast
Colorectal Cancer	Reactal Adenoma	1.9E-07	-2.675	39	Sabates-Bellver Colon
	Colon Adenoma	1.5E-07	-2.946	57	Sabates-Bellver Colon
	Reactal Adenoma	1E-16	-2.986	82	TCGA Colorectal
	Colon Adenocarcinoma	3.9E-16	-2.764	123	TCGA Colorectal
	Rectosigmoid Adenocarcinoma	0.00021	-2.325	25	TCGA Colorectal
Gastric Cancer	Diffuse Gastric Adenocarcinoma	6.4E-06	2.294	37	Derrico Gastric
	Clear Cell Renal Cell Carcinoma	0.00013	3.535	18	Lenburg Renal
	Papillary Renal Cell Carcinoma	6.2E-05	11.385	24	Yusenko Renal
	Clear Cell Renal Cell Carcinoma	0.00033	12.383	31	Yusenko Renal
Leukemia	T-Cell Childhood Acute Lymphoblastic Leukemia	0.00012	-3.373	50	Coustan-Smith Leukemia
Liver Cancer	Hepatocellular Carcinoma	5.3E-16	-2.492	180	Chen Liver
Lung Cancer	Large Cell Lung Carcinoma	9.9E-08	-4.172	84	Hou Lung
	Angioimmunpblastic T-cell Lymphoma	2.8E-05	2.169	26	Piccaluga Lymphoma

	Diffuse Large B-Cell Lymphoma	0.0002 7	-2.141	35	Alizadeh Lymphoma
	Anaplastic Large Cell Lymphoma	6.9E-0 5	-7.011	45	Eckerle Lymphoma
	Primary Cutaneous Anaplastic Large Cell	0.0003 8	-6.577	48	Eckerle Lymphoma
Other Cancer	Seminoma	9.2E-0 9	3.632	18	Korkola Seminoma
	Teratoma	9E-08	3.357	20	Korkola Seminoma
	Embtyonal Carcinoma	9.1E-0 8	5.268	21	Korkola Seminoma
	Mixed Germ Cell Tumor	4E-09	2.584	47	Korkola Seminoma
Sarcoma	Pleomorphic Liposarcoma	5.1E-0 8	5.516	18	Detwiller Sarcoma

Table S2. Summary of TCGA pan-cancer data

Primary Disease Type	TCGA ID	Total N	Primary Tumor	Normal Tissue
Adrenocortical carcinoma	ACC	79	79	0
Bladder Urothelial Carcinoma	BLCA	430	412	18
Breast invasive carcinoma	BRCA	1217	1105	112
Cervical squamous cell carcinoma and endocervical adenocarcinoma	CESC	309	306	3
Cholangiocarcino ma	CHOL	45	36	9
Colon adenocarcinoma	COAD	512	471	41
Lymphoid Neoplasm Diffuse Large B-cell Lymphoma	DLBC	48	48	0
Esophageal carcinoma	ESCA	173	162	11
Glioblastoma	GBM	173	168	5

multiforme				
Head and Neck squamous cell carcinoma	HNSC	546	503	43
Kidney Chromophobe	KICH	89	66	23
Kidney renal clear cell carcinoma	KIRC	607	535	72
Kidney renal papillary cell carcinoma	KIRP	321	289	32
Acute Myeloid Leukemia	LAML	151	151	0
Brain lower grade glioma	LGG	529	529	0
Liver hepatocellular carcinoma	LIHC	424	374	50
Lung adenocarcinoma	LUAD	585	526	59
Lung squamous cell carcinoma	LUSC	550	501	49
Mesothelioma	MESO	86	86	0
Ovarian serous cystadenocarcinoma	OV	379	379	0
Pancreatic adenocarcinoma	PAAD	182	178	4
Pheochromocytoma and paraganglioma	PCPG	186	183	3
Prostate adenocarcinoma	PRAD	551	499	52
Rectum adenocarcinoma	READ	177	167	10
Sarcoma	SARC	265	263	2
Skin cutaneous melanoma	SKCM	472	471	1
Stomach adenocarcinoma	STAD	407	375	32
Testicular germ cell tumor	TGCT	156	156	0
Thyroid carcinoma	THCA	568	510	58
Thymoma	THYM	121	119	2

Uterine corpus endometroid carcinoma	UCEC	583	548	35
Uterine carcinosarcoma	UCS	56	56	0
Uveal melanoma	UVM	80	80	0

Table S3. The list of the immune factors which significantly associated to APBB1IP expression

Type	Gene symbol	Definition
Immune inhibitory factors	TIGIT	Homo sapiens V-set and transmembrane domain containing 3 (VSTM3), mRNA.
	PDCD1LG2	Homo sapiens programmed cell death 1 ligand 2 (PDCD1LG2), mRNA.
	PDCD1	Homo sapiens spectrin, beta, non-erythrocytic 5 (SPTBN5), mRNA.
	LGALS9	Homo sapiens lectin, galactoside-binding, soluble, 9 (LGALS9), transcript variant 1, mRNA.
	LAG3	Homo sapiens lymphocyte-activation gene 3 (LAG3), mRNA.
	IL10	Homo sapiens interleukin 10 (IL10), mRNA.
	IDO1	Homo sapiens indoleamine-pyrrole 2,3 dioxygenase (INDO), mRNA.
	HAVCR2	Homo sapiens hepatitis A virus cellular receptor 2 (HAVCR2), mRNA.
	CTLA4	Homo sapiens cytotoxic T-lymphocyte-associated protein 4 (CTLA4), transcript variant 1, mRNA.
	CSF1R	Homo sapiens colony stimulating factor 1 receptor, formerly McDonough feline sarcoma viral (v-fms) oncogene homolog (CSF1R), mRNA.
	CD96	Homo sapiens CD96 molecule (CD96), transcript variant 2, mRNA.
	CD244	Homo sapiens CD244 molecule, natural killer cell receptor 2B4 (CD244), mRNA.
	BTLA	Homo sapiens B and T lymphocyte associated (BTLA), mRNA.
Immune stimulatory factors	TNFSF14	Homo sapiens tumor necrosis factor (ligand) superfamily, member 14 (TNFSF14), transcript variant 1, mRNA.
	TNFSF13B	Homo sapiens tumor necrosis factor (ligand) superfamily, member 13b (TNFSF13B), mRNA.
	TNFRSF9	Homo sapiens tumor necrosis factor receptor superfamily, member 9 (TNFRSF9), mRNA.
	TNFRSF17	Homo sapiens tumor necrosis factor receptor superfamily, member 17 (TNFRSF17), mRNA.
	TNFRSF13B	Homo sapiens tumor necrosis factor receptor superfamily, member 13B (TNFRSF13B), mRNA.
	LTA	Homo sapiens lymphotoxin alpha (TNF superfamily, member 1) (LTA), mRNA.
	IL6	Homo sapiens interleukin 6 (interferon, beta 2) (IL6), mRNA.

IL2RA	Homo sapiens interleukin 2 receptor, alpha (IL2RA), mRNA.
ICOS	Homo sapiens inducible T-cell co-stimulator ligand (ICOSLG), mRNA.
ENTPD1	Homo sapiens ectonucleoside triphosphate diphosphohydrolase 1 (ENTPD1), transcript variant 2, mRNA.
CXCR4	Homo sapiens chemokine (C-X-C motif) receptor 4 (CXCR4), transcript variant 2, mRNA.
CXCL12	Homo sapiens chemokine (C-X-C motif) ligand 12 (stromal cell-derived factor 1) (CXCL12), transcript variant 2, mRNA.
CD86	Homo sapiens CD86 molecule (CD86), transcript variant 2, mRNA.
CD80	Homo sapiens CD80 molecule (CD80), mRNA.
CD48	Homo sapiens CD48 molecule (CD48), mRNA.
CD40LG	Homo sapiens CD40 ligand (CD40LG), mRNA.
CD40	Homo sapiens CD40 molecule, TNF receptor superfamily member 5 (CD40), transcript variant 2, mRNA.
CD28	Homo sapiens CD28 molecule (CD28), mRNA.
CD27	Homo sapiens CD27 molecule (CD27), mRNA.
VSIR	Homo sapiens chromosome 10 open reading frame 54 (C10orf54), mRNA.

Table S4. The analysis of Gene Ontology (GO) terms mainly enriched in immune factors

Category	Term	Count	%	PValue	Genes	FDR
GOTERM_BP_ALL	GO:0051249~regulation of lymphocyte activation	17	5.5921	1.18E-24	IL2RA, TNFRSF13B, CTLA4, KLRK1, TNFSF14, IDO1, CD40, PDCD1LG2, IL10, TIGIT, BTLA, CD86, TNFSF13B, CD80, CD27, LAG3, CD28	1.81E-21
GOTERM_BP_ALL	GO:0002694~regulation of leukocyte activation	17	5.5921	8.01E-24	IL2RA, TNFRSF13B, CTLA4, KLRK1, TNFSF14, IDO1, CD40, PDCD1LG2, IL10, TIGIT, BTLA, CD86, TNFSF13B, CD80, CD27, LAG3, CD28	1.22E-20
GOTERM_BP_ALL	GO:0050865~regulation of cell activation	17	5.5921	1.92E-23	IL2RA, TNFRSF13B, CTLA4, KLRK1, TNFSF14, IDO1, CD40, PDCD1LG2, IL10, TIGIT, BTLA, CD86, TNFSF13B, CD80, CD27, LAG3, CD28	2.94E-20
GOTERM_BP_ALL	GO:0002682~regulation of immune system process	19	6.25	2.97E-21	IL2RA, TNFRSF13B, KLRK1, CTLA4, TNFSF14, IDO1, CD40, CXCL12, PDCD1LG2, IL10, TIGIT, BTLA, CD86, TNFSF13B, CD80, CD40LG, CD27, LAG3, CD28	4.54E-18
GOTERM_BP_ALL	GO:0050863~regulation of T cell activation	14	4.6053	4.63E-20	IL2RA, CTLA4, KLRK1, TNFSF14, IDO1, IL10, PDCD1LG2, TIGIT, BTLA, CD86, TNFSF13B, CD80, LAG3, CD28	7.08E-17

GOTERM_BP_ALL	GO:0002376~immune system process	23	7.5658	1.29E-19	IL2RA, TNFRSF13B, KLRK1, CTLA4, TNFRSF17, TNFSF14, CD40, CXCL12, PDCD1LG2, IL10, PDCD1, CD48, BTLA, CD96, CD86, TNFSF13B, CD80, CXCR4, CD40LG, ICOS, LTA, CD27, CD28	1.97E-16
GOTERM_CC_ALL	GO:0009897~external side of plasma membrane	14	4.6053	2.57E-18	CD244, IL2RA, TNFRSF13B, CTLA4, KLRK1, CD40, PDCD1, CD48, BTLA, CD86, CD80, ICOS, LAG3, CD28	2.51E-15
GOTERM_BP_ALL	GO:0006955~immune response	19	6.25	9.87E-17	IL2RA, TNFRSF13B, CTLA4, TNFSF14, TNFRSF17, CXCL12, PDCD1LG2, IL10, PDCD1, BTLA, CD96, CD86, TNFSF13B, CXCR4, CD40LG, ICOS, LTA, CD27, CD28	1.67E-13
GOTERM_CC_ALL	GO:0009986~cell surface	15	4.9342	8.20E-16	CD244, IL2RA, TNFRSF13B, CTLA4, KLRK1, CD40, PDCD1, CD48, TIGIT, BTLA, CD86, CD80, ICOS, LAG3, CD28	7.66E-13
GOTERM_BP_ALL	GO:0050670~regulation of lymphocyte proliferation	11	3.6184	7.63E-16	BTLA, IL2RA, TNFSF13B, CD80, TNFRSF13B, CTLA4, IDO1, CD40, PDCD1LG2, IL10, CD28	1.19E-12

Table S5. The analysis of Kyoto Encyclopedia of Genes and Genomes (KEGG) pathway mainly enriched in immune factors

Category	Term	Count	%	PValue	Genes	FDR
GOTERM_BP_ALL	GO:0051249 ~regulation of lymphocyte activation	17	5.59	1.18E-24	IL2RA, TNFRSF13B, CTLA4, KLRK1, TNFSF14, IDO1, CD40, PDCD1LG2, IL10, TIGIT, BTLA, CD86, TNFSF13B, CD80, CD27, LAG3, CD28	1.81E-21
GOTERM_BP_ALL	GO:0002694 ~regulation of leukocyte activation	17	5.59	8.01E-24	IL2RA, TNFRSF13B, CTLA4, KLRK1, TNFSF14, IDO1, CD40, PDCD1LG2, IL10, TIGIT, BTLA, CD86, TNFSF13B, CD80, CD27, LAG3, CD28	1.22E-20
GOTERM_BP_ALL	GO:0050865 ~regulation of cell activation	17	5.59	1.92E-23	IL2RA, TNFRSF13B, CTLA4, KLRK1, TNFSF14, IDO1, CD40, PDCD1LG2, IL10, TIGIT, BTLA, CD86, TNFSF13B, CD80, CD27,	2.94E-20

					LAG3, CD28	
GOTERM _BP_ALL	GO:0002682 ~regulation of immune system process	19	6.25	2.97E-21	IL2RA, TNFRSF13B, KLRK1, CTLA4, TNFSF14, IDO1, CD40, CXCL12, PDCD1LG2, IL10, TIGIT, BTLA, CD86, TNFSF13B, CD80, CD40LG, CD27, LAG3, CD28	4.54E-18
GOTERM _BP_ALL	GO:0050863 ~regulation of T cell activation	14	4.61	4.63E-20	IL2RA, CTLA4, KLRK1, TNFSF14, IDO1, IL10, PDCD1LG2, TIGIT, BTLA, CD86, TNFSF13B, CD80, LAG3, CD28	7.08E-17
GOTERM _BP_ALL	GO:0002376 ~immune system process	23	7.57	1.29E-19	IL2RA, TNFRSF13B, KLRK1, CTLA4, TNFRSF17, TNFSF14, CD40, CXCL12, PDCD1LG2, IL10, PDCD1, CD48, BTLA, CD96, CD86, TNFSF13B, CD80, CXCR4, CD40LG, ICOS, LTA, CD27, CD28	1.97E-16
GOTERM _CC_ALL	GO:0009897 ~external side of plasma membrane	14	4.61	2.57E-18	CD244, IL2RA, TNFRSF13B, CTLA4, KLRK1, CD40, PDCD1, CD48, BTLA, CD86, CD80, ICOS, LAG3, CD28	2.51E-15
GOTERM _BP_ALL	GO:0006955 ~immune response	19	6.25	9.87E-17	IL2RA, TNFRSF13B, CTLA4, TNFSF14, TNFRSF17, CXCL12, PDCD1LG2, IL10, PDCD1, BTLA, CD96, CD86, TNFSF13B, CXCR4, CD40LG, ICOS, LTA, CD27, CD28	1.67E-13
GOTERM _CC_ALL	GO:0009986 ~cell surface	15	4.93	8.20E-16	CD244, IL2RA, TNFRSF13B, CTLA4, KLRK1, CD40, PDCD1, CD48, TIGIT, BTLA, CD86, CD80, ICOS, LAG3, CD28	7.66E-13
GOTERM _BP_ALL	GO:0050670 ~regulation of lymphocyte proliferation	11	3.62	7.63E-16	BTLA, IL2RA, TNFSF13B, CD80, TNFRSF13B, CTLA4, IDO1, CD40, PDCD1LG2, IL10, CD28	1.19E-12

Table S6. Association between the clinicopathologic parameters and the risk score levels in GSE29016

Clinical variables	Total	Risk score levels		p value
		Low	High	
Gender				0.143
Male	30	18	12	
Female	38	16	22	
Age(years)				0.779
≤60	17	9	8	

>60	51	25	26	
Smoking				1
NO	16	8	8	
YES	52	26	26	
T Stage				
T1	21	12	9	0.431
T2+T3+T4	47	22	25	
N STAGE				0.231
N0	61	32	29	
>N0	7	2	5	
TNM Stage				0.225
I	40	21	19	
II+III+IV	70	45	25	
**TNM stage**				**0.038**
I	46	27	19	
II + III+ IV	22	7	15	
EGFR status				0.303
WT	64	31	33	
MUT	4	3	1	
KRAS status				0.203
WT	56	26	30	
MUT	12	8	4	
